# Differential effect of GLUT1 overexpression on survival and tumor immune microenvironment of human papilloma virus type 16-positive and -negative cervical cancer

**DOI:** 10.1038/s41598-019-49928-x

**Published:** 2019-09-16

**Authors:** Byoung Hyuck Kim, Ji Hyun Chang

**Affiliations:** 1 Department of Radiation Oncology, Seoul Metropolitan Government Seoul National University Boramae Medical Center, Seoul, Korea; 2 Department of Radiation Oncology, Seoul National University Hospital, Seoul, Korea

**Keywords:** Cancer microenvironment, Cervical cancer

## Abstract

Glucose transporter-1 (GLUT1) has been proposed as a prognosticator in various cancers associated with therapeutic resistance and immune evasion; however little data is available on the role of GLUT1 in cervical cancer. Most cervical cancers are caused by human papilloma virus (HPV), but studies on the treatment response and prognosis depending on the HPV subtype, are conflicting. This hypothesis-generating study aims to investigate the prognostic impact of GLUT1 in cervical cancer, in conjunction with HPV subtype. Clinicopathologic factors, along with mRNA expression data were obtained using The Cancer Genome Atlas database. Tumor HPV status and immune cell scores were extracted from previous publications. In total, 298 patients were analyzed. High GLUT1 expression was associated with old age, squamous cell carcinoma, high tumor stage, pelvic lymph node metastases, and low hysterectomy rate. Multivariate survival analysis revealed that high GLUT1 expression (Hazard ratio (HR) 2.57, p = 0.002) and HPV16 subtype (HR 0.56, p = 0.033) were independent prognostic factors for overall survival. In the subgroup analysis, poor prognostic impact of high GLUT1 expression was maintained in HPV16-positive group (p < 0.001), but not in HPV16-negative group (p = 0.495). Decreased immune cell scores of CD8^+^ T cells, B cells, and Th1 cells by high GLUT1 expression were observed only in HPV16-positive group. In conclusion, these results suggested that GLUT1 expression and HPV16 subtype might have an independent prognostic value in cervical cancer. GLUT1-mediated immunomodulation might be an important cause of treatment failure, especially in HPV16-positive group.

## Introduction

Cervical cancer is still occurring worldwide at a considerable frequency despite significant advances in the screening of pre-invasive lesion, as well as modern prevention strategies with the high-risk human papilloma virus (HPV) vaccination^1^. Although HPV infection has been considered as an important cause of cervical cancer, studies on the treatment response and prognosis according to the different HPV subtypes are conflicting^2–4^. Several studies suggested that HPV genotypes might be associated with the prognosis of cervical cancer patients treated with radiotherapy^5,6^. In addition, cancer linked to the high-risk HPV is considered to be more immunogenic because of the production of viral proteins^7^. Although clinical stage, lymph node metastasis, lymphovascular invasion, and tumor size are commonly used to predict the prognosis, patients with similar prognostic factors often experience very different therapeutic responses in actual clinics. Further stratification based on novel biologic markers may help clinicians developing individualized strategies for heterogeneous group of patients.

HPV infection could promote certain cancer hallmarks including immune evasion and epithelial-mesenchymal transition, increasing the activity of glycolytic enzymes^8,9^. Altered tumor metabolic status has been recognized as an important contributing factor to chemo- or radio-resistance. Among the glycolytic enzymes, glucose transporter-1 (GLUT1), which facilitates glucose transport across the plasma membrane, has been reported to be a poor prognosticator in various cancers^10–12^. Previous meta-analysis suggested that the overexpression of GLUT1 was associated with enhanced invasiveness, proliferative potential, and decreased patients’ survival^13^. However, little data is available for the prognostic significance of GLUT1 in cervical cancer.

Therefore, to elucidate the effect of the combination of HPV genotypes and tumor metabolism, mediated by GLUT1, on the treatment outcomes and tumor microenvironment in cervical cancer, we analyzed these biomarkers in patients with cervical squamous cell carcinoma and endocervical adenocarcinoma (CESC), using The Cancer Genome Atlas (TCGA) database.

## Results

### Patients’ characteristics

Differential GLUT1 mRNA expression levels in tumor and normal tissues are shown according to TCGA classification in Supplemental Table 1. CESC ranked second among the pan-cancers in terms of GLUT1 gene differential fold change (tumor over normal tissues).

The characteristics of the patients included in this study are presented in Supplemental Table 2. Specifically, the mean age was 48 years (range 20–88 years). Squamous cell carcinoma was found in 247 (82.9%) patients, and adenocarcinoma was found in the remaining 51 patients (17.1%). About half of the patients had clinical stage I disease. The patients with diverse stage distribution received heterogeneous treatments including hysterectomy, radiotherapy, and/or chemotherapy. In terms of HPV infection, HPV16 was found in 164 (55.0%) patients, HPV18 in 39 (13.1%) patients, and other HPV subtypes in 70 (23.5%) patients. Only 18 patients were negative for all types of HPV (6.0%). Available follow-up data showed that recurrence occurred in 48 (16.1%) patients and death in 72 (24.2%) patients.

Upon subdivision of the patients into two groups based on the GLUT1 expression level, we found significant differences in characteristics (Table 1). High GLUT1 expression was associated with age ≥50, squamous cell carcinoma, high tumor stage, pelvic lymph node metastases, low tumor grade, and low hysterectomy rate. On the contrary, when the patients were divided into two groups based on HPV16 positivity, we did not find association with clinicopathologic factors, except for a low tumor grade and age <50 (Supplemental Table 3).

**Table 1:** Descriptive characteristics according to the GLUT1 mRNA expression level.

	GLUT1 low (n = 204)	GLUT1 high (n = 94)	p
Age ≥ 50			0.018
No	135 (66.2%)	48 (51.1%)	
Yes	69 (33.8%)	46 (48.9%)	
Histology			<0.001
Adenocarcinoma	49 (24.0%)	2 (2.1%)	
Squamous cell carcinoma	155 (76.0%)	92 (97.9%)	
Clinical stage			0.001
I	123 (61.8%)	35 (38.0%)	
II	42 (21.1%)	25 (27.2%)	
III	23 (11.6%)	22 (23.9%)	
IV	11 (5.5%)	10 (10.9%)	
Pelvic LN metastases			0.046
No	103 (71.5%)	28 (54.9%)	
Yes	41 (28.5%)	23 (45.1%)	
Hysterectomy			0.001
No	68 (33.3%)	52 (55.3%)	
Yes	136 (66.7%)	42 (44.7%)	
Grade ≥ 3			0.003
No	96 (50.8%)	56 (71.8%)	
Yes	93 (49.2%)	22 (28.2%)	
Radiotherapy			0.877
No	86 (42.2%)	38 (40.4%)	
Yes	118 (57.8%)	56 (59.6%)	
HPV16			0.184
Negative	93 (46.5%)	34 (37.4%)	
Positive	107 (53.5%)	57 (62.6%)	

LN, lymph node; HPV, human papilloma virus.

### Survival analysis

**Figure 1:**
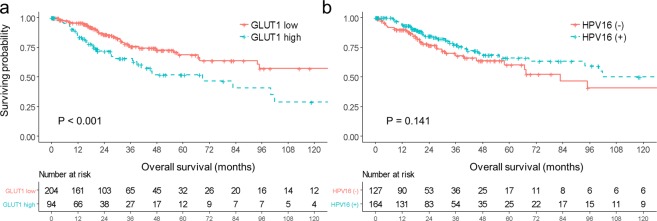
Kaplan-Meier curve of overall survival (**a**) by GLUT1 expression (**b**) by HPV16 positivity in all study patients.

The results of the univariate analyses for OS and PFS are listed in Table 2. Four factors appeared to be associated with OS in univariate analysis: clinical stage, pelvic lymph node (LN) metastases, hysterectomy, and GLUT1 expression. However, all factors except GLUT1 expression were excluded from the Cox proportional hazard model, due to the issue of multicollinearity in relation to GLUT1 expression (all p-value <0.05 by Pearson correlation test). Other than that, age, histology, and tumor grade were entered into the multivariate model to adjust the potential confounding effects, although they did not significantly affect OS in the univariate analysis. Multivariate survival analysis revealed that high GLUT1 expression (HR 2.57, p = 0.002) and HPV16 subtype (HR 0.56, p = 0.033) were independent prognostic factors for OS (Table 3). Kaplan-Meier curves of overall survival based on GLUT1 expression or HPV16 positivity in all study patients were shown in Fig. 1. Five-year OS rates were 51.7% in GLUT1-high group and 68.7% in GLUT1-low group (p < 0.001).

**Table 2:** Univariate analysis of prognostic factors for overall survival (OS) and progression-free survival (PFS).

Characteristics		No.	5 yr OS (%)	P	5 yr PFS (%)	P
Age (years)	<50	115	62.2	0.315	73.6	0.116
	≥50	183	64.4		61.3	
Histology	ADC	51	55.2	0.805	49.7	0.147
	SqCC	247	65.0		73.3	
Clinical Stage	I–II	225	66.6	0.001	68.3	0.299
	III–IV	66	49.2		69.6	
Pelvic LN metastases	No	131	76.7	0.008	75.6	0.032
	Yes	64	58.0		54.4	
Hysterectomy	No	120	53.9	0.003	72.4	0.781
	Yes	178	70.0		67.2	
Grade	1–2	152	64.2	0.563	69.6	0.152
	3–4	115	67.9		66.7	
Radiotherapy	No	124	57.9	0.935	72.1	0.224
	Yes	174	65.0		67.3	
HPV16 status	Negative	127	60.4	0.141	57.7	0.022
	Positive	164	66.2		77.6	
GLUT1 expression	Low	204	68.7	0.001	69.5	0.581
	high	94	51.7		67.1	

ADC, adenocarcinoma; SqCC, squamous cell carcinoma; LN, lymph node; HPV, human papilloma virus.

**Table 3:** Multivariate analysis of prognostic factors for overall survival.

Characteristics		HR	95% CI	P
GLUT1 expression	Low	1		
	High	2.566	1.404–4.688	0.002
HPV16	Negative	1		
	Positive	0.558	0.326–0.955	0.033
Age (years)	<50	1		
	≥50	0.921	0.542–1.564	0.760
Histology	Adenocarcinoma	1		
	Squamous cell carcinoma	0.562	0.276–1.141	0.111
Grade	1–2	1		
	3–4	0.942	0.530–1.673	0.838

HR, hazard ratio; CI, confidence interval; HPV, human papilloma virus.

On the other hand, HPV16 positivity and pelvic LN metastases were significant prognostic factors for PFS in univariate analysis. However, none of them maintained statistical significance in multivariate analysis.

### Exploratory subgroup analysis assessing the impact of GLUT1 on

**Figure 2:**
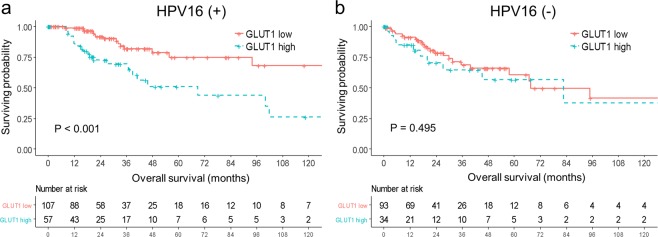
Kaplan-Meier curve of overall survival by GLUT1 expression level stratified with HPV16 status.

Supplemental Fig. 1 plotted HR and 95% CI after comparing OS based on the GLUT1 expression, between each patient subgroups. The significance of high GLUT1 expression varies depending upon several factors. Specifically, by focusing on HPV16 positivity, the poor prognostic impact of high GLUT1 expression was maintained only in HPV16-positive group (HR 3.326, 95% CI 1.700–6.509, p < 0.001), and not in HPV16-negative group (HR 1.293, 95% CI 0.617–2.708, p = 0.495). Survival curves based on GLUT1 expression and HPV16 status were shown in Fig. 2.

To further elucidate the cause of the differential impact of GLUT1 overexpression on OS between HPV16-positive and -negative patients, possible differences in the tumor immune microenvironment were investigated. As shown in Table 4, decreased immune cell scores of T cells, CD8^+^ T cells, B cells, Th1 cells, and exhausted CD8^+^ cells induced by high GLUT1 expression were observed only in the HPV16-positive group, whereas no significant differences was found in the HPV16-negative group.

**Table 4:** Immune cell scores according to the GLUT1 expression level, and those stratified with HPV16 status.

Cell type	Overall		p	HPV16 (+)		p	HPV16 (−)		p
	low (n = 204)	high (n = 94)		low (n = 107)	high (n = 57)		low (n = 93)	high (n = 34)	
T cells	6.0 ± 1.7	5.6 ± 1.3	0.009	6.2 ± 1.6	5.6 ± 1.2	0.010	5.8 ± 1.7	5.5 ± 1.5	0.324
CD8 T cells	7.3 ± 1.9	6.5 ± 1.6	<0.001	7.5 ± 1.8	6.4 ± 1.6	<0.001	7.2 ± 1.9	6.6 ± 1.6	0.089
B cells	3.5 ± 1.7	2.7 ± 1.5	<0.001	3.5 ± 1.7	2.6 ± 1.6	0.002	3.4 ± 1.7	2.9 ± 1.4	0.156
Th1 cells	4.5 ± 1.7	4.1 ± 1.4	0.034	4.6 ± 1.6	4.0 ± 1.3	0.015	4.4 ± 1.7	4.2 ± 1.5	0.742
Dendritic cells	4.7 ± 1.5	4.4 ± 1.1	0.149	4.5 ± 1.5	4.4 ± 1.2	0.470	4.8 ± 1.5	4.5 ± 1.0	0.180
Macrophages	7.6 ± 1.4	7.5 ± 1.2	0.735	7.6 ± 1.4	7.4 ± 1.2	0.406	7.5 ± 1.4	7.5 ± 1.1	0.935
Exhausted CD8	5.8 ± 1.3	5.5 ± 1.0	0.018	5.8 ± 1.2	5.4 ± 1.0	0.030	5.8 ± 1.3	5.6 ± 1.0	0.353
Neutrophils	4.1 ± 1.2	4.1 ± 1.0	0.703	4.0 ± 1.1	4.1 ± 1.0	0.970	4.3 ± 1.3	4.1 ± 1.0	0.593
Cytotoxic cells	6.8 ± 1.8	6.5 ± 1.5	0.129	6.9 ± 1.6	6.5 ± 1.4	0.153	6.7 ± 1.8	6.3 ± 1.7	0.327
Treg cells	5.9 ± 1.5	5.7 ± 1.2	0.280	6.1 ± 1.3	5.8 ± 1.1	0.095	5.7 ± 1.6	5.6 ± 1.4	0.888
NK CD56dim cells	2.8 ± 1.4	2.5 ± 1.0	0.100	2.8 ± 1.3	2.5 ± 1.0	0.143	2.8 ± 1.5	2.5 ± 1.0	0.279
Mast cells	5.6 ± 1.8	5.5 ± 1.7	0.507	5.8 ± 1.8	5.6 ± 1.6	0.500	5.5 ± 1.8	5.4 ± 1.8	0.745
NK cells	3.2 ± 1.4	3.0 ± 1.3	0.334	3.2 ± 1.4	3.0 ± 1.4	0.367	3.2 ± 1.3	3.1 ± 1.2	0.738

## Discussion

In the present study, we demonstrated that high GLUT1 expression and HPV16 subtype are independent prognostic factors in cervical cancer. Interestingly, poor prognostic impact as well as differences in the tumor immune microenvironment linked to high GLUT1 expression were observed only in HPV16-positive group.

GLUTs are plasma membrane transporters that facilitate glucose transport across the cell membrane. Upregulation of GLUTs is present in most cancer cells and can be one of the initial carcinogenic changes^14^. Among GLUT family, it is known that high GLUT1 expression level is associated with advanced tumor stage, squamous histology, high tumor grade, depth of invasion, and poor differentiation in various cancers^10,11,15,16^. Our results also validated these findings in TCGA cervical cancer cohort.

Until now, few studies have evaluated the prognostic value of GLUT1 in cervical cancer reporting conflicting results. Kim *et al*. investigated hypoxia and metabolic markers in cervical cancer demonstrating that the high expression of HIF-1α and c-Met was associated with low OS, whereas the high expression of GLUT1 and CA9 did not show significant impact on survival^17^. On the contrary, Kanjanapan *et al*. recently showed that high GLUT1 expression was associated with low PFS (HR 2.8, p = 0.049) and OS (HR 5.0, p = 0.011) in 60 patients with locally advanced cervical cancer^18^, and these findings are consistent with our study. These previous studies were based on immunohistochemistry results, which might be subjective, therefore may produce different results. In our study, we quantitatively assessed GLUT1 mRNA expression level since there was no GLUT1 protein expression data in the TCGA database.

We hypothesized that one of the reasons of the above conflicting results for the prognostic role of GLUT1 in cervical cancer may result from the difference in the proportion of HPV16-positive patients. In our study, HR for high GLUT1 expression was 3.326 in the HPV16-positive group (p < 0.001) and 1.293 in the HPV16-negative group (p = 0.495), showing that the role of GLUT1 may differ depending on HPV16 status. Our study is the first report suggesting a differential prognostic impact of GLUT1 according to the carcinogenic virus type. Carcinogenic viruses can regulate tumor metabolic profiles via direct or indirect interaction with GLUT1^19^. According to above findings, HPV16-related cancer may be more addicted to the glycolysis pathway through its oncoprotein E6/E7, thus GLUT1 expression level could have an important prognostic value in HPV-positive cervical cancer^20,21^. HPV16 E7 protein can alter the activity of pyruvate kinase, which enables deriving metabolic energy mostly from glycolytic processes rather than from oxidative phosphorylation^20^. Moreover, the expression changes of E6/E7 significantly promote the expression of hypoxia-inducible factor 1-alpha (HIF-1α) which activates the transcription of transporters and enzymes regulating the glycolysis and the pentose phosphate shunt^21^. On the other hand, HPV16-negative tumors may have an alternative metabolic dependency. For example, HPV18 cancers have higher unspliced transcripts of the E6 oncoprotein than HPV16 cancers, suggesting different functional implications of E6 and E7, according to the HPV genotypes^22^. Determining unrevealed prognostic determinants of HPV16-negative cervical cancer would be critical in developing further strategies for enhancing treatment response.

Previous studies estimated that HPV16 accounts for about half of all cervical cancers and HPV18 accounts for an additional 15%, although there are at least 15 known oncogenic HPV types^4,23,24^. These proportions are very similar to our patient characteristics shown in Supplemental Table 2, and this similarities support our presumptive methods’ relevance. Several studies suggested that HPV types might be a useful prognostic factor in cervical cancer. Ghong *et al*. reported that patients with HPV16-positive cancer had better diseaes-free survival (HR 0.41; p = 0.0019)^24^. Schwartz *et al*. demonstrated that patients with HPV18-related cancers showed higher cervical cancer specific mortality (HR 2.5) compared with those with HPV16-related cancers^3^. Similarly, we found that HPV16 subtype was an independent prognostic factor for OS after adjusting for clinical factors in the multivariate analysis. The reason why HPV16 subtype is associated with a better OS is largely unknown. However, it has been suggested that HPV16-positive cells have increased sensitivity to cancer therapies, a higher level of tumor apoptosis, a slower growth rate, and an enhanced immune response towards the virus in several cancer cells, providing a possible explanation for the increased radiation sensitivity of cervical cancer with HPV16 positivity^25,26^.

To further investigate the phenotype of GLUT1 overexpression in HPV16-positive cervical cancer, we examined the effects of GLUT1 overexpression on tumor immune microenvironment, based on the hypothesis that GLUT1-mediated immune evasion in the HPV16-positive cervical cancer could affect the treatment outcome^27,28^. Our results revealed that individual immune cell scores of T cells, CD8^+^ T cells, and B cells were significantly reduced in high GLUT1 expression group of HPV16-positive cervical cancer. It is known that metabolic perturbations within tumor induced by high GLUT1 level modulate tumor immune microenvironment by suppressing T cells; this effect is due to a metabolic competition and to the generation of metabolic byproducts^29,30^. GLUT1-mediated immune escape from cytotoxic T cells of tumor cells may be the cause of reduced survival.

Our results raise the possibility of considering GLUT1 as a potential novel target of HPV16-induced cervical cancer. Similar to the immune checkpoint blockade, strategies to reverse GLUT1-mediated immune escape could also be considered as potential treatment. Clinically, GLUT1 has not been directly targeted since specific and potent inhibitors are still not found^14^. Zhao *et al*. have shown that the inhibition of GLUT1 by the small-molecule inhibitor WZB117, could increase the radiosensitivity of breast cancer cells^31^. Other novel inhibitors of GLUT1 are under development^14^.

The present study has several limitations. First, there is a possibility that the integration of HPV information and the immune cell scores from different studies may be an obstacle in the interpretation of the main grouping, despite they all used the same TCGA data. Second, the generalization of our findings could be restricted by the lack of a suitable external validation cohort. Lastly, the relatively small number of patients as well as the heterogeneity of the treatment modalities might also have affected the prognostic impact of the analyzed variables.

## Conclusions

Despite of these limitations, our results suggested that GLUT1 expression and HPV16 subtype might have an independent prognostic value in cervical cancer. This study will provide new hypothesis and strategies for the sensitization of cancer cells to radiotherapy or chemotherapy through the regulation of glucose metabolism. Pre-treatments testing the HPV status may make personalized treatment possible. GLUT1-mediated immunomodulation might be an important cause of treatment failure, especially in the HPV16-positive group; thus, the incorporation of GLUT1 inhibitors may be helpful to increase treatment efficacy. Further proof-of-concept study is warranted to identify novel immunometabolic targets to overcome therapeutic resistance.

## Methods

### Patient population and data acquisition

All methods were in accordance with the relevant guidelines and regulations, and were approved by the Ethical Review Committee of the Seoul Metropolitan Government Seoul National University Boramae Medical Center. As the data were de-identified in the TCGA database, the ethics committee approved that the requirement of informed consent was waived. This study complied with TCGA publication guidelines and policies (http://cancergenome.nih.gov/publications/publicationguidelines). Clinicopathologic factors and mRNA expression data were obtained from TCGA database, using TCGA-biolinks package^32^. Clinical information including tumor stage, tumor histology, disease extent, and treatment course were collected and analyzed. In total, 298 cervical cancer patients with appropriate survival data were included. The sample types of the included patients were all primary solid tumors except for two metastatic tissues.

### HPV status and genotype

Tumor HPV status and genotype were determined according to the supplemental information from a previous TCGA publication, which calculated them using MassArray and RNA-sequencing^22^. For patients who did not fulfill the inclusion criteria for the above study, their HPV status and genotype were determined using another previous publication from TCGA, which used the number of normalized reads mapping to each HPV reference (DNA cut-off of 50 normalized counts of HPV aligned reads)^33^.

### Immune cell scores

Immune cell scores were extracted from a previous TCGA study, which calculated these scores in 9986 RNASeq samples from 24 different tumor types^34^. Briefly, each immune cell score was calculated from specific 60 marker genes, whose expression levels could classify 14 immune cell populations. These results were highly reproducible and concordant with flow cytometry and immunohistochemistry results^34^.

### Statistical analysis

To compare the clinicopathologic features between two groups, student’s t-test and chi-square test were applied to continuous variables and categorical variables, respectively. Using clinical follow-up data version 4.0, the overall survival (OS) and the progression-free survival (PFS) were defined from the date of initial diagnosis to the date of death or the last follow-up and to the date of disease progression, respectively. Survival estimates were calculated by the Kaplan-Meier method and compared using the log-rank test. The dichotomous cut-off point discriminating the OS between GLUT1-high and -low patients, was determined by the maximal chi-square test, which divided all the patients into two groups based on the median z-score of 0.165. Statistically significant factors in univariate analysis were checked for collinearity, using Pearson correlation coefficient. Factors with a p-value < 0.05 on univariate analysis or factors considered clinically important (e.g., HPV16 genotype) were subjected to the multivariate analysis. In multivariate analysis, Cox proportional hazards regression models were applied to adjust the potential confounding effects of associated variables and also were used to calculate hazard ratios (HR) and 95% confidence intervals (CI). R 3.3.0 statistical package (https://www.r-project.org/) was used for all statistical analyses.

## Supplementary information

Supplemental tables 1–3 and figure 1

dataset 1

## Data Availability

The raw datasets of the current study are available in the Supplemental Dataset.
