# Supplementary material for: Differential effect of GLUT1 overexpression on survival and tumor immune microenvironment of human papilloma virus type 16-positive and -negative cervical cancer
Source: Sci Rep. 2019 Sep 16;9:13301. doi: 10.1038/s41598-019-49928-x (PMC6746783; doi:10.1038/s41598-019-49928-x)

Differential effect of GLUT1 overexpression on survival and tumor immune microenvironment of human papilloma virus type 16-positive and -negative cervical cancer

Byoung Hyuck Kim, Ji Hyun Chang*

Department of Radiation Oncology, Seoul Metropolitan Government Seoul National University Boramae Medical Center, Seoul, Korea.

Supplemental Table 1. Differential expression of GLUT1 gene between tumor and normal tissue in pan-TCGA cohort data

Cancer type	Acronym	Fold change (tumor over normal)	P value	Number of normal sample	Number of cancer sample	Mean GLUT1 expression _normal	Mean GLUT1 expression _cancer
Lung squamous cell carcinoma	LUSC	5.4262	0.000	51	502	8.5722	13.9984
Cervical and endocervical cancers	CESC	3.6195	0.035	3	307	9.63	13.2495
Cholangiocarcinoma	CHOL	3.0629	0.000	9	36	6.2852	9.3481
Lung adenocarcinoma	LUAD	2.9903	0.000	59	530	8.1978	11.1881
Esophageal carcinoma	ESCA	2.5382	0.002	11	185	10.299	12.8372
Kidney renal clear cell carcinoma	KIRC	1.9257	0.000	72	538	10.4614	12.3871
Head and Neck squamous cell carcinoma	HNSC	1.8871	0.000	44	522	12.4095	14.2966
Uterine Corpus Endometrial Carcinoma	UCEC	1.7834	0.000	35	550	10.2105	11.9939
Pancreatic adenocarcinoma	PAAD	1.6193	0.133	4	179	10.4727	12.092
Rectum adenocarcinoma	READ	1.5543	0.001	10	167	10.7328	12.2871
Colon adenocarcinoma	COAD	1.4003	0.000	41	475	10.6645	12.0648
Sarcoma	SARC	1.2842	0.217	2	263	8.5505	9.8347
Breast invasive carcinoma	BRCA	1.2641	0.000	114	1116	9.65	10.9141
Stomach adenocarcinoma	STAD	0.8267	0.002	35	415	10.3646	11.1913
Bladder urothelial carcinoma	BLCA	0.8051	0.123	19	411	12.0611	12.8662
Glioblastoma multiforme	GBM	0.5861	0.006	5	168	11.1848	11.7709
Thyroid carcinoma	THCA	0.4733	0.000	59	513	9.3539	9.8272
Liver hepatocellular carcinoma	LIHC	0.3676	0.017	50	374	6.8533	7.2209
Pheochromocytoma and Paraganglioma	PCPG	0.1766	0.478	3	184	8.5823	8.7589
Thymoma	THYM	-0.1552	0.597	2	120	9.3285	9.1733
Prostate adenocarcinoma	PRAD	-0.3457	0.002	52	502	10.4402	10.0945
Kidney renal papillary cell carcinoma	KIRP	-0.3803	0.017	32	291	10.3906	10.0103
Kidney Chromophobe	KICH	-0.9116	0.000	25	66	10.6418	9.7302

Supplemental Table 2. Overall patients characteristics (N=298)

Characteristics		No. of patients (%)
Mean age, years		48.3 +/- 13.9
Race	White	204 (68.5%)
	Others	59 (19.8%)
	NA	35 (11.7%)
Histology	Squamous cell carcinoma	247 (82.9%)
	Adenocarcinoma	51 (17.1%)
Clinical stage	I	158 (53.0%)
	II	67 (22.5%)
	III	45 (15.1%)
	IV	21 (7.0%)
	NA	7 (2.3%)
Pelvic LN metastasis	Yes	64 (21.5%)
	No	131 (44.0%)
	NA	103 (34.6%)
Distant metastasis	M1	10 (3.4%)
	M0 or Mx	288 (96.6%)
Hysterectomy	Yes	178 (59.7%)
	No	120 (40.3%)
Histologic grade	G1	18 (6.0%)
	G2	134 (45.0%)
	G3	114 (38.3%)
	G4	1 (0.3%)
	NA	31 (10.4%)
LVI	Yes	75 (25.2%)
	No	70 (23.5%)
	NA	153 (51.3%)
Radiotherapy	Yes	174 (58.4%)
	No	124 (41.6%)
Sample type	Primary	296 (99.4%)
	Metastatic	2 (0.6%)
HPV subtype	16	164 (55.0%)
	18	39 (13.1%)

	Others	70 (23.5%)
	Negative	18 (6.0%)
	NA	7 (2.3%)
Recurrence	Yes	48 (16.1%)
Death	Yes	72 (24.2%)

NA, non-available; LN, lymph node; LVI, lymphovascular invasion; HPV, human papilloma virus.

Supplemental Table 3. Descriptive characteristics according to the HPV16 positivity

HPV16	Negative (N=127)	Positive (N=164)	p
Age≥50			0.003
No	65 (51.2%)	113 (68.9%)	
Yes	62 (48.8%)	51 (31.1%)	
Histology			0.699
Adenocarcinoma	24 (18.9%)	27 (16.5%)	
Squamous cell carcinoma	103 (81.1%)	137 (83.5%)	
Clinical stage			0.810
I	63 (50.8%)	90 (56.2%)	
II	30 (24.2%)	36 (22.5%)	
III	21 (16.9%)	24 (15.0%)	
IV	10 (8.1%)	10 (6.2%)	
Pelvic LN metastases			0.308
No	53 (72.6%)	74 (64.3%)	
Yes	20 (27.4%)	41 (35.7%)	
Hysterectomy			0.141
No	59 (46.5%)	61 (37.2%)	
Yes	68 (53.5%)	103 (62.8%)	
Grade≥3			0.001
No	50 (44.6%)	99 (66.9%)	
Yes	62 (55.4%)	49 (33.1%)	
Radiotherapy			0.511
No	50 (39.4%)	72 (43.9%)	
Yes	77 (60.6%)	92 (56.1%)	
GLUT1 expression			0.184
Low	93 (73.2%)	107 (65.2%)	
High	34 (26.8%)	57 (34.8%)	

LN, lymph node; HPV, human papilloma virus

Supplemental Figure 1. Hazard ratio and 95% confidence interval for overall survival according to the GLUT1 expression level in the subgroup of patients for each characteristic

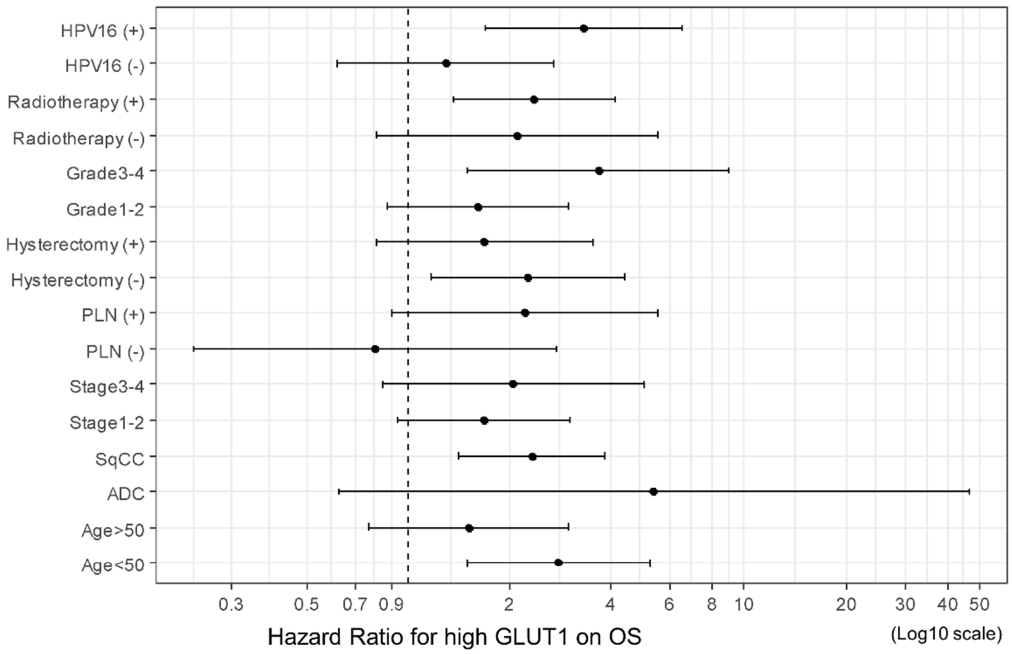

Supplement: Supplementary file 1 — Supplemental tables 1–3 and figure 1 [file 41598_2019_49928_MOESM1_ESM.pdf]
